# Development and Validation of a Nomogram-Based Model to Predict Primary Hypertension Within the Next Year in Children and Adolescents: Retrospective Cohort Study

**DOI:** 10.2196/58686

**Published:** 2024-12-30

**Authors:** Chenlong Qin, Li Peng, Yun Liu, Xiaoliang Zhang, Shumei Miao, Zhiyuan Wei, Wei Feng, Hongjian Zhang, Cheng Wan, Yun Yu, Shan Lu, Ruochen Huang, Xin Zhang

**Affiliations:** 1 Department of Medical Informatics, School of Biomedical Engineering and Informatics Nanjing Medical University Nanjing China; 2 Institute of Medical Informatics and Management Nanjing Medical University Nanjing China; 3 Department of Endocrinology and Metabolism, the Fourth Affiliated Hospital Nanjing Medical University Nanjing China; 4 Department of Information, the First Affiliated Hospital Nanjing Medical University Nanjing China; 5 Suqian Hospital, Jiangsu Province Hospital Suqian China; 6 Women and Children Department of the First Affiliated Hospital of Nanjing Medical University Nanjing China

**Keywords:** independent risk factors, prediction model, primary hypertension, clinical applicability, development, validation, pediatrics, electronic health records

## Abstract

**Background:**

Primary hypertension (PH) poses significant risks to children and adolescents. Few prediction models for the risk of PH in children and adolescents currently exist, posing a challenge for doctors in making informed clinical decisions.

**Objective:**

This study aimed to investigate the incidence and risk factors of PH in Chinese children and adolescents. It also aimed to establish and validate a nomogram-based model for predicting the next year’s PH risk.

**Methods:**

A training cohort (n=3938, between January 1, 2008, and December 31, 2020) and a validation cohort (n=1269, between January 1, 2021, and July 1, 2023) were established for model training and validation. An independent cohort of 576 individuals was established for external validation of the model. The result of the least absolute shrinkage and selection operator regression technique was used to select the optimal predictive features, and multivariate logistic regression to construct the nomogram. The performance of the nomogram underwent assessment and validation through the area under the receiver operating characteristic curve, concordance index, calibration curves, decision curve analysis, clinical impact curves, and sensitivity analysis.

**Results:**

The PH risk factors that we have ultimately identified include gender (odds ratio [OR] 3.34, 95% CI 2.88 to 3.86; *P*<.001), age (OR 1.11, 95% CI 1.08 to 1.14; *P*<.001), family history of hypertension (OR 42.74, 95% CI 23.07 to 79.19; *P*<.001), fasting blood glucose (OR 6.07, 95% CI 4.74 to 7.78; *P*<.001), low-density lipoprotein cholesterol (OR 2.03, 95% CI 1.60 to 2.57; *P*<.001), and uric acid (OR 1.01, 95% CI 1.01 to 1.01; *P*<.001), while factor breastfeeding (OR 0.04, 95% CI 0.03 to 0.05; *P*<.001) has been identified as a protective factor. Subsequently, a nomogram has been constructed incorporating these factors. Areas under the receiver operating characteristic curves of the nomogram were 0.892 in the training cohort, 0.808 in the validation cohort, and 0.790 in the external validation cohort. Concordance indexes of the nomogram were 0.892 in the training cohort, 0.808 in the validation cohort, and 0.790 in the external validation cohort. The nomogram has been proven to have good clinical benefits and stability in calibration curves, decision curve analysis, clinical impact curves, and sensitivity analysis. Finally, we observed noteworthy differences in uric acid levels and family history of hypertension among various subgroups, demonstrating a high correlation with PH. Moreover, the web-based calculator of the nomogram was built online.

**Conclusions:**

We have developed and validated a stable and reliable nomogram that can accurately predict PH risk within the next year among children and adolescents in primary care and offer effective and cost-efficient support for clinical decisions for the risk prediction of PH.

## Introduction

In the past 20 years, primary hypertension (PH) has become the dominant cause of arterial hypertension (AH) in children aged older than 6 years, especially in adolescents [1]. The American Heart Association suggested that pediatric PH is a condition that is severely underrecognized in the latest scientific statement [2]. In an analysis of a large pediatric health care claims database, PH was found to be approximately 10 times more prevalent than secondary hypertension (SH; 0.2% versus 0.02%) [3]. PH in children and adolescents is the early phase of a condition that exists on a continuum across the life course, with higher BP exposure over time contributing to subclinical outcomes in childhood and CVD events later in life [2,3]. However, the diagnosis of PH is a diagnosis of exclusion, the etiology of PH has not been elucidated yet [2].

For newly diagnosed children and adolescents with AH, screening for PH is challenging and time-consuming. According to the latest guideline proposed by a consensus panel composed of multiple institutions such as the European Society of Cardiology Associations and Councils, the differential diagnosis between primary and SH should include detailed family history, physical examination, and laboratory tests [4]. In case of abnormal examination results, the consensus panel agrees that further diagnostic investigations are needed [4]. This contributes to a time-consuming and costly diagnostic process for PH. The current situation is that inexperienced clinicians cannot make suspected diagnoses to recommend proper examinations and tend to recommend too many examinations, which leads to a waste of medical resources and delayed intervention measures [5]. Therefore, the American Heart Association recommends using clinical decision-support tools for detecting hypertension in children and adolescents to achieve the goal of simple, accurate, and cost-effective risk prediction [2,6,7].

However, all of the following studies have the same deficiencies [8-10]. (1) Neglecting PH: the above studies focused on the disease of AH in children and adolescents, neglecting that PH has become the predominant subtype of AH in children and adolescents over the past two decades, with a more complex diagnostic process and severe long-term effects on organs in adulthood. (2) Inadequate use of electronic health record (EHR) data: the risk prediction models discussed in the above studies were developed using survey data and ignored that EHR data encompasses a broader array of both confirmed and potential PH-related risk factors, which allows for a more accurate reflection of the physical state of children and adolescents. This omission could be one of the reasons behind the suboptimal prediction results observed in the prediction models. (3) None of the aforementioned studies have undergone external validation or prospective validation, which significantly impairs the usability of the prediction model.

To address the above limitations, we analyzed the medical condition of PH in children and adolescents and aimed to develop an accurate, rapid, and cost-effective nomogram to predict their PH risk within the next year based on their EHR data. The nomogram was trained and validated on the data of the First Affiliated Hospital of Nanjing Medical University and performed external validation on the data of the Fourth Affiliated Hospital of Nanjing Medical University and the Suqian Hospital. Additionally, we have developed a free online tool implementing nomograms to assist doctors in accurate and low-cost identification of high-risk populations for PH in a timely manner.

## Methods

### Study Population and Data Collection

**Figure 1:**
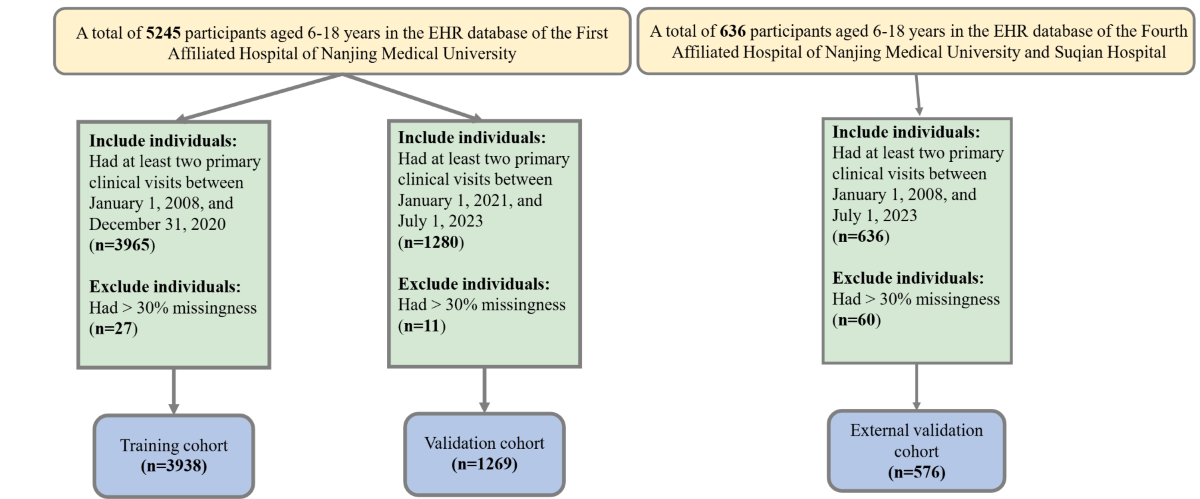
Flowchart of the procedure. A total of 5245 participants aged 6-18 years in the EHR database of the First Affiliated Hospital of Nanjing Medical University from January 1, 2008, to July 1, 2023, were enrolled in this study. A total of 3938 participants were finally enrolled in the training cohort to construct the nomogram and 1269 participants were finally enrolled in the validation cohort for nomogram validation, respectively. A total of 576 participants aged 6-18 years in the EHR database of the Fourth Affiliated Hospital of Nanjing Medical University and the Suqian Hospital from January 1, 2008, to July 1, 2023, were finally enrolled for independent external validation. EHR: electronic health record.

Data from the EHR database of the First Affiliated Hospital of Nanjing Medical University were used for model development. As shown in Figure 1, we identified a cohort of patients aged 6-18 years who had at least two primary clinical visits between January 1, 2008, and July 1, 2023 (5245 participants). Patients were excluded if they had any diagnosis of hypertension, pregnancy, or were using antihypertensive drugs at cohort entry. Participants who had >30% missingness (38 participants) were also excluded. Overall, a total of 5207 participants were finally enrolled after rigorous screening. For the prediction modeling cohort, cases in the training population referred to patients from January 1, 2008, to December 31, 2020 (3938 participants), whereas cases in the validation cohort were patients from January 1, 2021, to July 1, 2023 (1269 participants), and were used for model validation.

An independent cohort from the Fourth Affiliated Hospital of Nanjing Medical University and the Suqian Hospital was used for external validation. In brief, we screened a total of 53 children and adolescents with PH and 523 children and adolescents without PH from the EHR databases of these two hospitals using the same screening process as when modeling.

The demographic and clinical parameters from all the EHR databases were collected, including diagnosis of PH, gender, age, family history of hypertension, family history of diabetes, breastfeeding situation, white blood cell count, lymphocyte count (LYC), lymphocyte percentage (LYP), platelet distribution (PDW), mean platelet volume, plateletcrit, platelet count, red blood cell distribution width-coefficient of variation, mean cell hemoglobin concentration, mean corpuscular hemoglobin, mean corpuscular volume (MCV), hematocrit, hemoglobin, red blood cell count, basophil count, basophil percentage, eosinophil count, eosinophil percentage, neutrophil count, neutrophil percentage (NEUTP), monocyte count (MONOC), monocyte percentage, retinol-binding protein, kalium, natrium, chlorine, calcium, phosphorus, magnesium, fasting blood glucose (FBG), creatinine, urea, uric acid (UA), total protein, albumin, triglycerides, aspartate aminotransferase, alanine aminotransferase, lactate dehydrogenase, γ-glutamyl transpeptidase, alkaline phosphatase, total bilirubin, direct bilirubin, indirect bilirubin (IBIL), total cholesterol (TC), high-density lipoprotein cholesterol, low-density lipoprotein cholesterol (LDL-C), lipoprotein(a), creatine kinase, adenosine deaminase, white/bulb ratio, globulin, α-hydroxybutyrate dehydrogenase. The family history of diabetes and hypertension was defined by the reported history of diabetes and hypertension in immediate family members.

For participants with an outcome event, the index date is defined as the date of the first PH diagnosis. For those without an outcome event, the index date is set as the date of the last non-PH diagnosis. The EHR data from all participants within the year preceding the respective index date is averaged for model training, validation, and external validation.

### Ethical Considerations

Our study was performed per the Transparent Reporting of a Multivariable Prediction Model for Individual Prognosis or Diagnosis statement [11]. All procedures were reviewed and approved by the Ethics Committee of Nanjing Medical University (2023-SR-500). Written informed consent for participation was not required for this study per the national legislation and the institutional requirements. All personal privacy information was well protected and removed during the process of analysis and publication.

### Data Preprocessing

A total of 58 variables were reviewed (Table S1 in Multimedia Appendix 1), and 14 variables with missing data exceeding 20% were excluded from the analysis. To address the missing data for the remaining 44 variables, we used chained random forests with predictive mean matching [12]. After imputation, continuous variables were transformed into standardized scores (*z* scores) by subtracting their respective means and dividing by their SDs. Finally, we apply the RandomOverSampler algorithm to the training dataset to solve the problem of sample imbalance [13].

### Definition of PH

Our outcome was PH, with the adoption of the *ICD-10-CM* (*International Classification of Diseases, 10th Revision, Clinical Modification*) since October 1, 2015, the diagnosis of PH was defined using the *ICD-10-CM* category I10.

### Statistical Analysis

Continuous variables following a normal distribution were reported as means (SD) and analyzed with the Student *t* test (2-tailed) to assess differences between the training and validation cohorts. For continuous variables with skewed distributions, they were described as median (25th percentile, 75th percentile) and analyzed with the Mann-Whitney *U* test. Categorical data were represented as numbers (percent) and analyzed through the chi-square test or Fisher exact test to make comparisons.

We first used the least absolute shrinkage and selection operator (Lasso) regression technique to identify significant features in the training cohort, which can be obtained from the R (R Foundation) package *glmnet* [14]. Then, the multivariable logistic regression model was constructed through sequentially selected candidate variables in the Lasso regression. Predictors were eliminated using the forward stepwise regression strategy based on their respective *P* values to construct the final model [15]. The nomogram was developed using factors that exhibited a two-sided *P* value <.05 in the multivariate analysis, implemented by the R package *rms*. We used the area under the receiver operating characteristic curve (AUC) and concordance index (C-index) to assess the predictive accuracy and discriminatory ability of the model using the R package *pROC* and *Hmisc*, respectively. The calibration curve was used to evaluate the consistency between nomogram-predicted and observed risks. The decision curve analysis (DCA) was conducted to gauge the net benefit of identifying true high-risk patients that ought to have intervention and the net reduction of unnecessary interventions using the R package *rmda*. The clinical utility of the nomogram was also measured by the clinical impact curves for a population size of 1000. Stratified analyses were displayed by forest plots using the R package *forestploter*.

Statistical analyses were performed with R software (version 4.2.3; RRID:SCR_001905). Two-sided *P* value <.05 was considered to be statistically significant.

## Results

### Baseline Characteristics

A total of 5207 participants from the EHR database of the First Affiliated Hospital of Nanjing Medical University were finally enrolled after rigorous screening. Table S1 in Multimedia Appendix 2 shows the baseline characteristics of participants in the training and validation cohort. For model training, 3938 participants between January 1, 2008, and December 31, 2020, were used to build the training cohort, and 1269 participants between January 1, 2021, and July 1, 2023, were used to build the validation cohort for model validation. The total prevalence of PH in the whole cohort was 10% (521 participants). The training cohort consisted of 3938 participants, while the validation cohort had 1269 participants assigned to it. The prevalence of PH in the training cohort was 9.8% (385 participants) and 10.7% (136 participants) in the validation cohort, respectively. Of the training cohort, the median age of children and adolescents was 14 years with a range from 6 to 17, and 15 years with a range from 6 to 17 in the validation cohort. More than half of the participants were girls both in the training cohort (55.51%, 2186 participants) and validation cohort (54.5%, 692 participants). There were no significant differences in the characteristics of diagnosis of PH, family history of hypertension, family history of diabetes, gender, age, albumin, calcium, FBG, hematocrit, hemoglobin, IBIL, LDL-C, LYC, LYP, MCV, MONOC, NEUTP, PDW, red blood cell count, TC, triglycerides, UA, and urea between 2 cohorts.

A total of 576 participants from the EHR database of the Fourth Affiliated Hospital of Nanjing Medical University and the Suqian Hospital were used for external validation. There were 53 participants with PH and 523 participants without PH in the external validation cohort. Table S2 in Multimedia Appendix 1 shows the baseline characteristics of participants in the training cohort and external validation cohort.

### Development and Construction of Nomogram

**Figure 2:**
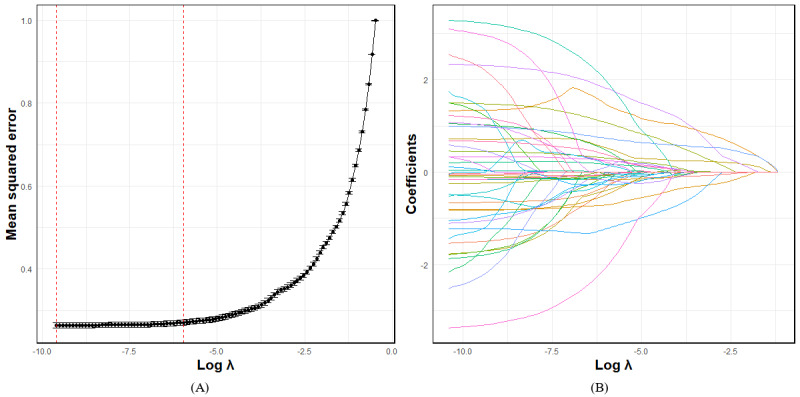
Predictor selection using the Lasso binary logistic regression model. (A) The optimal penalization coefficient λ in the Lasso model was identified through 10-fold cross-validation in the training cohort. (B) Lasso coefficient profiles of all predictors. The trajectory of each coefficient related to PH features was observed within the Lasso coefficient profiles as the λ parameter changed in the Lasso algorithm. Lasso: least absolute shrinkage and selection operator.

According to the Lasso regression model, 38 candidate predictors had nonzero coefficients including gender, age, family history of hypertension, family history of diabetes, breastfeeding, PDW, mean platelet volume, plateletcrit, platelet count, red blood cell distribution width-coefficient of variation, mean cell hemoglobin concentration, MCV, hematocrit, hemoglobin, neutrophil count, NEUTP, MONOC, monocyte percentage, LYC, LYP, kalium, natrium, calcium, FBG, creatinine, UA, albumin, lactate dehydrogenase, total bilirubin, direct bilirubin, TC, high-density lipoprotein cholesterol, LDL-C, lipoprotein(a), white/bulb ratio, globulin, IBIL, and creatine kinase (Figures 2A and 2B). Out of these candidate predictors, we used the forward stepwise regression strategy to construct the final multivariate logistic regression model, which revealed that gender, age, family history of hypertension, breastfeeding, FBG, LDL-C, and UA were independent risk factors for PH (Table 1). Using the coefficient estimates from this final logistic model, nomogram was defined as:

Nomogram_Score_ = –257.5045 + 45.4545 × FBG + 17.8043 × LDL–C + 0.2351 × UA + 30.3525 × Gender + 2.5831 × AGE + 94.6279 × Family history of hypertension – 83.3383 × Breastfeeding

**Figure 3:**
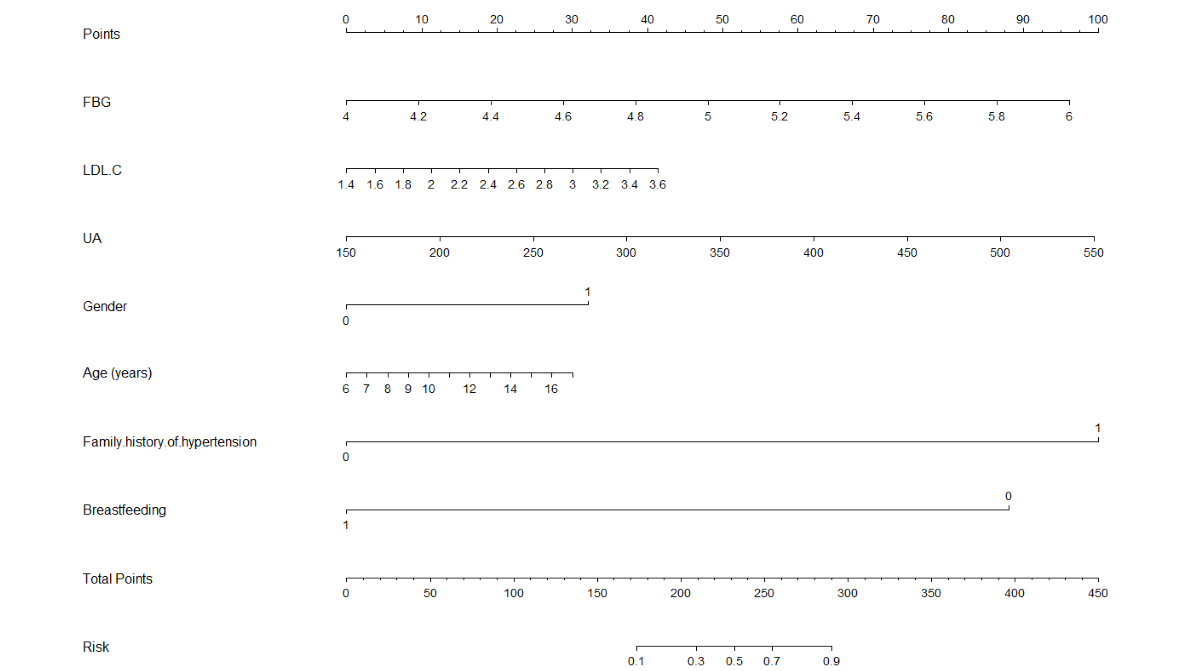
Nomogram for the prediction of primary hypertension. To use the nomogram, add up the scores for each feature of the participants to calculate the total score. Then, draw a vertical line at the corresponding position on the total score axis to determine the associated risk of PH. For example, a girl aged 10 years with a family history of hypertension and a history of breastfeeding, FBG 6 mmol/L, LDL-C 2 mmol/L, and UA 400 umol/L has a total score of 10+0+95+0+91+10+59=265. This indicates that the risk of developing PH is 83%. FBG: fasting blood glucose; LDL-C: low-density lipoprotein cholesterol; PH: primary hypertension; UA: uric acid.

These 7 independent factors were used to construct the nomogram (Figure 3).

**Table 1:** Risk factors for primary hypertension in the training cohort.

Predictors		β coefficient	Odds ratio (95% CI)	*P* values
**Family history of hypertension**				
	No	Reference	—^a^	—
	Yes	3.76	42.74 (23.07 to 79.19)	<.001
**Breastfeeding**				
	No	Reference	—	—
	Yes	–3.31	0.04 (0.03 to 0.05)	<.001
**Gender**				
	Female	Reference	—	—
	Male	1.2	3.34 (2.88 to 3.86)	<.001
	Age (years)	0.1	1.11 (1.08 to 1.14)	<.001
	Fasting blood glucose	1.8	6.07 (4.74 to 7.78)	<.001
	Low-density lipoprotein cholesterol	0.71	2.03 (1.6 to 2.57)	<.001
	Uric acid	0.01	1.01 (1.01 to 1.01)	<.001

^a^Not applicable.

### Assessment of the Nomogram

**Figure 4:**
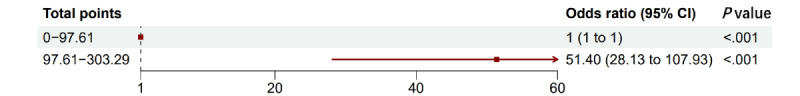
Association between the total points of the nomogram and primary hypertension.

**Figure 5:**
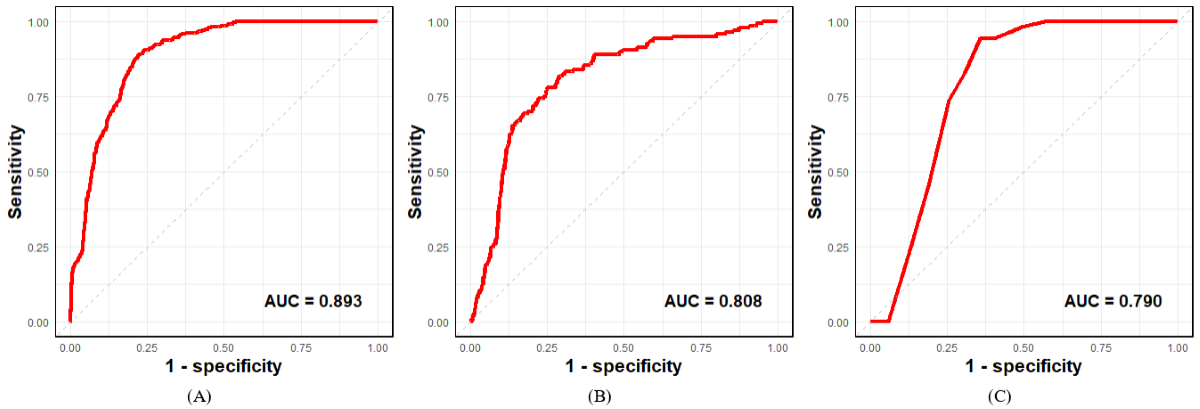
ROC curves for the prediction of primary hypertension in the training, validation, and external validation cohort. (A) ROC curves of the factors and nomogram in the training cohort. (B) ROC curves of the factors and nomogram in the validation cohort. (C) ROC curves of the factors and nomogram in the external validation cohort. AUC: area under the receiver operating characteristic curve; ROC: receiver operating characteristic.

**Figure 6:**
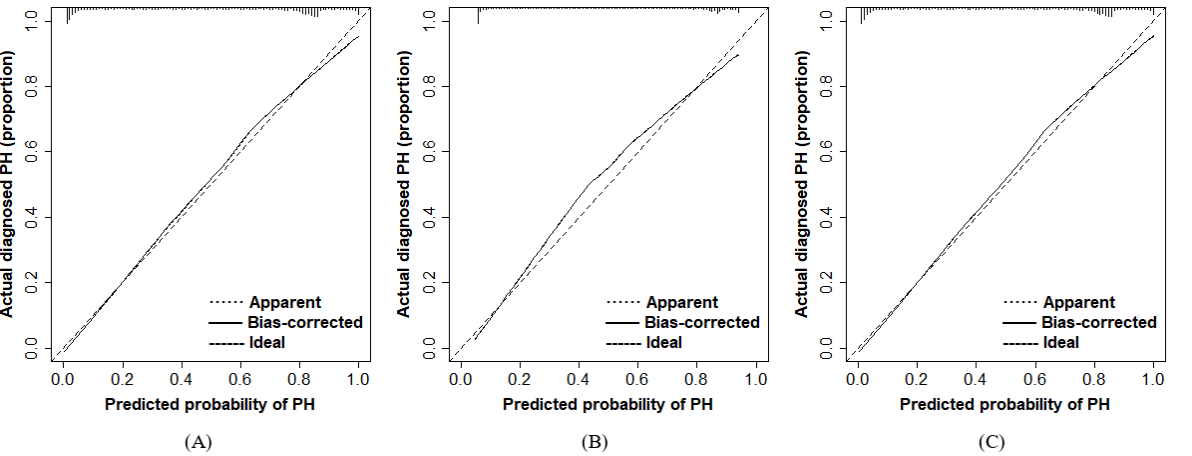
Calibration curves of the nomogram prediction in the training, validation, and external validation cohort. (A) Calibration curves of nomogram prediction in the training cohort. (B) Calibration curves of nomogram prediction in the validation cohort. (C) Calibration curves of nomogram prediction in the external validation cohort. PH: primary hypertension.

The nomogram was constructed to predict the risk of PH in children and adolescents by using gender, age, family history of hypertension, breastfeeding, FBG, LDL-C, and UA. First, we calculated the total nomogram points for each participant and divided the points into two subgroups according to the median. Figure 4 shows that the risk of PH increased with the total nomogram points, and participants in the high point subgroup (total points: 97.61-303.29) had a significantly higher PH risk than those in the low point subgroup. We next constructed the AUC and C-index to assess the performance of the model. The AUC value of the nomogram in the training cohort was 0.892 (95% CI 0.884-0.899), and the AUCs of gender, age, family history of hypertension, breastfeeding, FBG, LDL-C, and UA were 0.692 (95% CI 0.681-0.702), 0.744 (95% CI 0.733-0.756), 0.555 (95% CI 0.549-0.560), 0.735 (95% CI 0.727-0.744), 0.567 (95% CI 0.553-0.581), 0.670 (95% CI 0.657-0.684), and 0.782 (95% CI 0.771-0.794), respectively (Figure 5A). The C-index of the nomogram was 0.892 (95% CI 0.884-0.899) in the training cohort, signifying its robust predictive discriminatory ability. Moreover, the calibration curve of the training cohort revealed a high consistency between prediction and actual observation (Figure 6A).

### Validation of the Nomogram

The data of the validation cohort were used to independently validate the nomogram. In the validation cohort, the AUC value of the nomogram was 0.808 (95% CI 0.769-0.846; Figure 5B), and the AUC of UA was 0.742 (95% CI 0.692-0.791). The nomogram exhibited a C-index of 0.808 (95% CI 0.770-0.846) in the validation cohort, indicating commendable predictive discrimination within the model. However, the calibration curve revealed a relatively modest consistency between predictions and actual observations (Figure 6B).

We have performed an external validation of the nomogram using an independent cohort. The AUC value of the nomogram was 0.790 (95% CI 0.751-0.830), and the C-index of the nomogram was 0.790 (95% CI 0.764-0.849) in the external validation (Figure 5C). Moreover, the calibration curve revealed a high consistency between prediction and actual observation in the external validation (Figure 6C).

### Clinical Net Benefits With Nomogram

**Figure 7:**
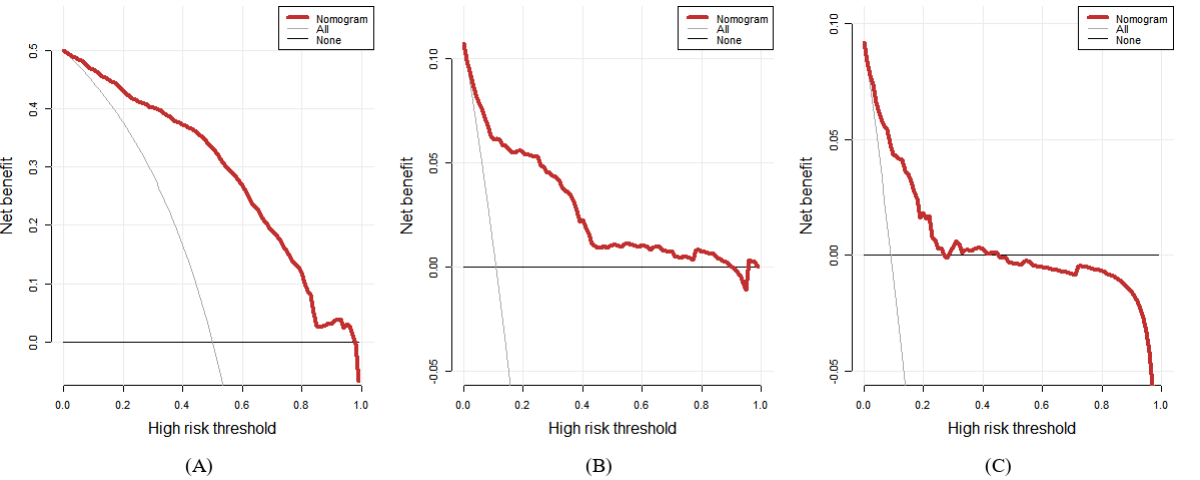
DCA of the nomogram prediction in the training, validation, and external validation cohort. (A) DCA of nomogram prediction in the training cohort. (B) DCA of nomogram prediction in the validation cohort. (C) DCA of nomogram prediction in the external validation cohort. DCA: decision curve analysis.

**Figure 8:**
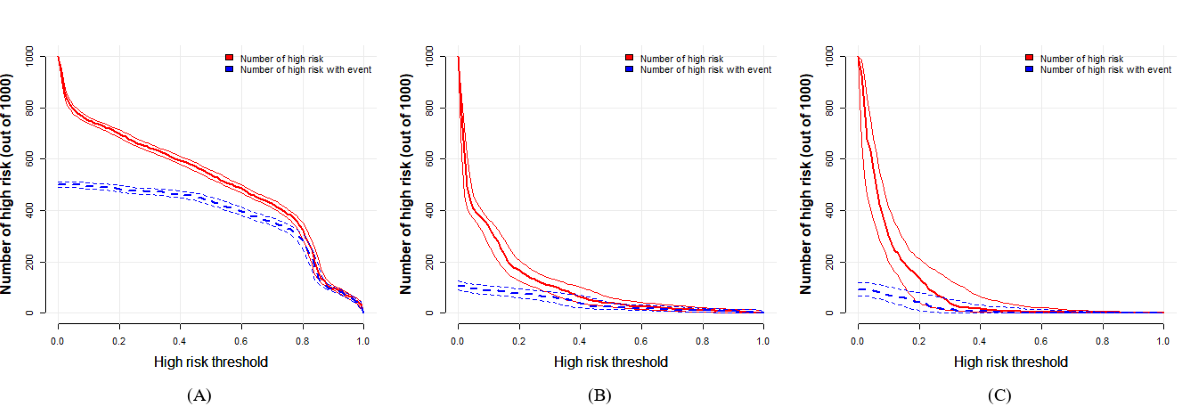
Clinical impact curves of the nomogram prediction in the training, validation, and external validation cohort. (A) Clinical impact curves of nomogram prediction in the training cohort. (B) Clinical impact curves of nomogram prediction in the validation cohort. (C) Clinical impact curves of nomogram prediction in the external validation cohort.

The DCA illustrated that the nomogram conferred more clinical net benefits than several competing intervention strategies, namely, intervention for all and intervention for none, as depicted in Figure 7A. Specifically, with a threshold probability of 0.8, the nomogram presented the best net benefit. Similar findings were observed in the validation cohort represented in Figure 7B and the external validation cohort represented in Figure 7C. The clinical impact curves for the nomogram revealed a close alignment between predicted and actual probabilities in the training cohort, as illustrated in Figure 8A. Comparable results were observed in the validation cohort (Figure 8B) and the external validation cohort (Figure 8C).

### Sensitivity Analysis of Nomogram Prediction

**Figure 9:**
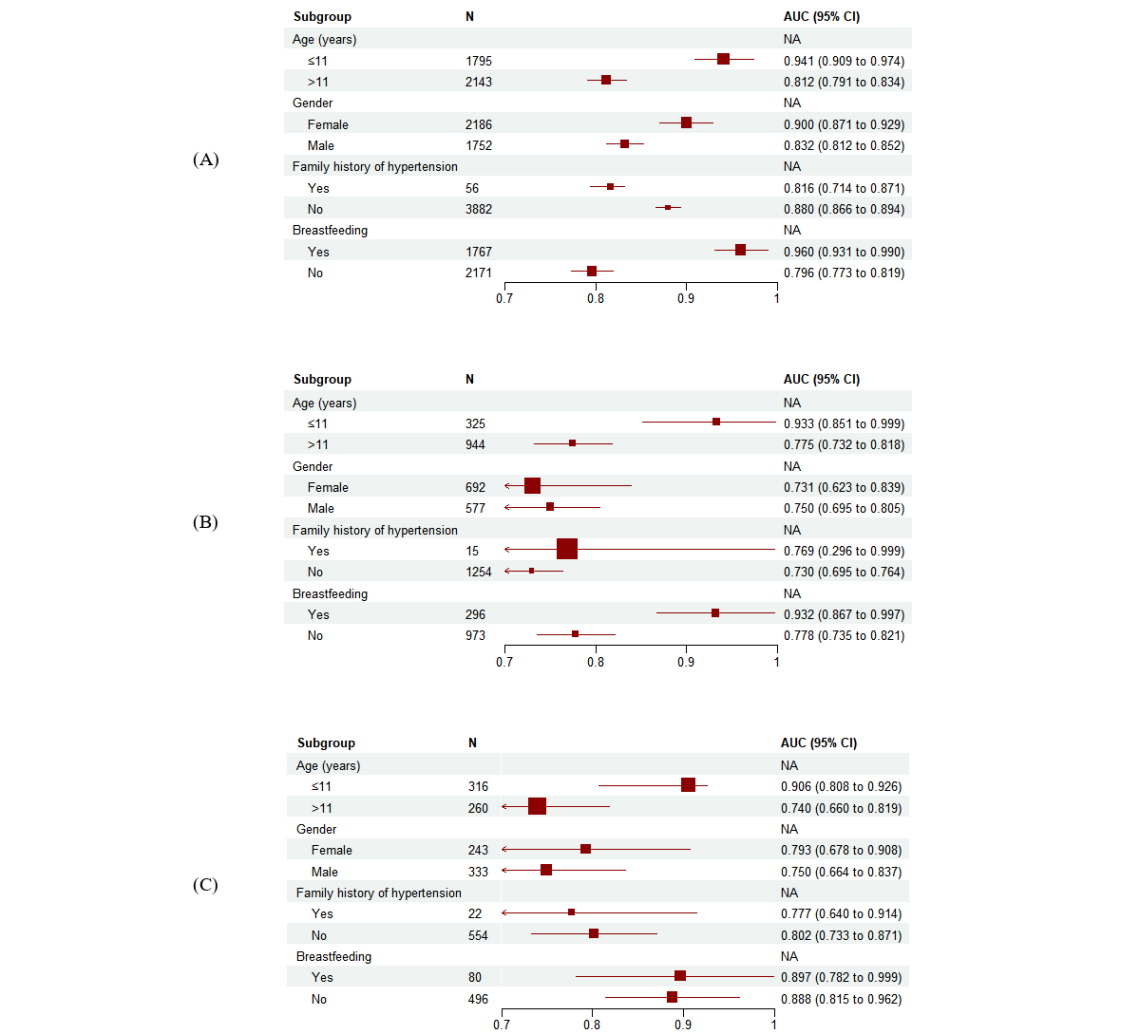
Subgroup analyses of nomogram. (A) Prediction accuracy for PH in the training cohort. (B) Prediction accuracy for PH in the validation cohort. (C) Prediction accuracy for PH in the external validation cohort. PH: primary hypertension.

To assess the robustness of the nomogram, we performed a series of subgroup analyses with subgroups defined by age, gender, and PH status. The nomogram had reasonable AUCs in all of these subpopulations, ranging from 0.796 (95% CI 0.773-0.819) to 0.960 (95% CI 0.931-0.990) in the training cohort, 0.730 (95% CI 0.695-0.764) to 0.933 (95% CI 0.851-0.999) in the validation cohort, and 0.716 (95% CI 0.694-0.832) to 0.900 (95% CI 0.808-0.926) in the external validation cohort (Figures 9A-9C). It is worth noting that the nomogram performed better in the population aged 11 years or younger than in the population aged older than 11 years in the sensitivity analysis across all three cohorts.

### Website of Nomogram

A web-based nomogram calculator for PH has been developed and is freely accessible online [16]. This tool is designed to provide diagnostic probability, facilitating guardians and physicians in the user-friendly identification of PH among children and adolescents.

## Discussion

### Principal Results

In this study, we have constructed and validated a risk prediction model of future 1-year incident PH in children and adolescents. The EHR-based nomogram achieved 0.892, 0.808, and 0.790 AUCs in training, validation, and external validation cohorts, respectively. Moreover, the DCA and clinical impact curve indicated that a substantial proportion of the threshold probabilities in this model yielded favorable net benefits. In sensitivity analysis, the nomogram showed relatively stable and consistent results in subgroups of age, gender, and PH status, making it the most sensitive predictive tool for PH risk while ensuring accuracy.

Accurately, timely, and low-cost identification of children and adolescents at high risk of PH is of great significance. Previous studies have preliminarily constructed risk prediction models targeting the hazards of AH [8-10]. However, as the leading type of AH in children and adolescents, the differential diagnosis of PH is more challenging and time-consuming. PH children are highly likely to become PH adults and to have measurable target organ injury if timely intervention is not applied. It is therefore suggested to build a model to identify children and adolescents at high risk of PH and intervene promptly to minimize harm as much as possible.

Our study differed from previous research in that we constructed a nomogram using EHR data to achieve initial screening of children and adolescents at high PH risk. Compared to survey data, EHR data encompasses a broader array of both confirmed and potential PH-related risk factors, which allows for a more accurate reflection of the physical state of children and adolescents. Furthermore, EHR data is readily accessible in clinical settings, providing a reliable and objective source of information unaffected by subjective influences from patients. Meanwhile, EHR data can be shared among different medical institutions, which is beneficial for the external validation of predictive models. The application of the EHR-based nomogram in primary care institutions can effectively improve the diagnosis of pediatric PH, reducing its difficulty and time consumption. This suggests that our model can serve as a clinical decision-support tool to assist clinicians in screening children and adolescents with high PH risk.

We used Lasso as a variable selection technique to reduce the number of parameters, thereby reducing the overfitting and complexity of the model, making it easier to interpret, and reducing operational complexity. Previous studies have demonstrated that Lasso regression is a widely used method for high dimensional predictor selection and nomogram construction [10,17], it shrinks the size of the coefficients of the independent variables depending on their predictive power. Some coefficients may shrink down to zero, allowing us to restrict the model to variables with nonzero coefficients. In our study, we calculated the mean squared error for each variable individually. The λ that provided the minimum mean squared error (minMSE) on the data was 0.00007 and the λ with the minMSE + 1 SE of minMSE (minMSE + 1 SE) was 0.00308. Considering that after the value of λ reached a certain threshold, further increasing the number of independent variables in the model did not significantly improve the model’s performance. Therefore, we decided to use the minMSE + 1 SE criterion to select the best predictive feature. This approach allowed us to maintain model performance while simultaneously reducing model complexity and mitigating overfitting.

The nomogram incorporated 7 parameters including gender, age, family history of hypertension, breastfeeding, FBG, LDL-C, and UA. Consistent with prior research findings, our study also identified gender and age as potential high-risk factors for PH [1,18,19]. Notably, there was a more pronounced sex-related disparity, indicating a higher ratio of boys to girls (3-4:1) among adolescents with PH (Figure S1 in Multimedia Appendix 1). To delve deeper into the variations in PH influenced by gender and age, we conducted a subgroup analysis. We first categorized the participants into two subgroups based on their PH status both in the training cohort and validation cohort (Table S3 in Multimedia Appendix 1). In the training cohort, males constituted a substantial majority among PH patients, comprising 77.7% (299 participants), in stark contrast to females who accounted for only 22% (86 participants). Additionally, individuals without PH were generally younger than their counterparts with PH. These trends were consistently observed in the validation cohort. Then, we divided the participants into two subgroups using the age of 11 years as the cutoff point in the training and validation cohorts (Table S4 in Multimedia Appendix 1). In the training cohort, diagnoses with PH were recorded for 1% (n=25) of participants aged 11 years or younger and 16.8% (360 participants) of participants aged older than 11 years, the results in the validation cohort are approximately similar. Finally, we divided both the training and validation cohorts into male and female subgroups based on gender (Table S5 in Multimedia Appendix 1). In the training cohort, participants with PH were recorded for 4% (86 participants) of females and 17.1% (299 participants) of males, and 3% (21 participants) of females and 19.9% (115 participants) of males in the validation cohort, respectively.

We observed significant differences in UA levels among different subgroups in all 3 subgroup analyses. Accumulating evidence supports the notion that elevated UA levels in children and adolescents are an independent risk factor for hypertension. In 2000, Fujiwara et al [20] confirmed that elevated UA levels are a typical characteristic of PH in children and adolescents. A recent report from the Study of High Blood Pressure in Pediatrics, Adult Hypertension Onset in Youth highlighted that mean UA concentrations increased from 5.3 to 5.9 mg/dL as blood pressure values escalated from below the 80th to above the 90th percentile [21]. Moreover, a cohort study with an average follow-up time of 5.7 years also confirmed that higher baseline UA levels increased the risk of incident hypertension (hazard ratio 1.19, 95% CI 1.03-1.38) [22]. This aligns with the results observed in our research, further reinforcing the association between elevated UA levels and an increased risk of PH in children and adolescents.

Our research findings indicate that children and adolescents who were breastfed exhibit a lower risk of PH compared to those who were not breastfed. Several studies have demonstrated that breastfeeding, considered an environmental factor, exerts a protective effect on blood pressure [23-25]. Moreover, our study revealed that children and adolescents with a positive family history of hypertension face an elevated risk of developing PH. While there is a widespread consensus on the genetic factors contributing to PH in children and adolescents, the specific underlying mechanism still requires further research [26-28]. In our study, we observed a noteworthy escalation in the risk of PH among children and adolescents with rising levels of FBG. The reason may be that insulin resistance and hyperinsulinemia can trigger the activation of the sympathetic nervous system, representing key mechanisms associated with the pathophysiology of obesity-related hypertension in children and adolescents [29-32].

Evidence has been raised that childhood obesity is linked not only to the onset of PH during childhood but also to an elevated risk of cardiovascular disease in adulthood [33-36]. Giussani et al [37] also confirmed elevated BMI is the most potent risk factor for PH in children and adolescents in one of their studies. In 2012, an analysis included data from 63,025 overweight and obese children and adolescents found that high LDL-C levels are related to an increased BMI. In 2012, an analysis incorporating data from 63,025 overweight and obese children and adolescents revealed an association between elevated levels of LDL-C with increased BMI [38]. In our research, we observed a correlation between obesity and PH in children and adolescents. Our findings indicated that with the elevation of LDL-C values, the risk of PH in children and adolescents also increased. This further substantiates the robust connection between obesity and PH in this demographic.

Importantly, all of these predictors are routinely accessible through standard clinical examinations. This implies that the model we have established in our study could serve as a potentially valuable tool for the rapid assessment of PH in children and adolescents, surpassing the capabilities of previous models. In the macro-view, identifying the PH predictors for youths would relieve the burden of monitoring and administering youth health and economizing facilities and resources. Furthermore, these identifications can facilitate the implementation of early interventions for those at risk. These interventions encompass a range of strategies, including but not limited to instilling disease awareness in children and adolescents from an early age and preemptively averting the transition to other potential hazards, such as adverse cardiovascular events.

### Limitations

There are also some limitations in our study. First, due to various factors such as cuff size, poor compliance, and rapid changes in the body during growth, medical institutions often struggle to record accurate BP in children and adolescents [39-42]. Bearing this in mind, we decided not to include BP in our study. Second, as previously mentioned, distinguishing between PH and SH can be challenging; thus, it is possible that some individuals diagnosed with SH may have been inadvertently included in this study. Moreover, in practical applications, the model faces limitations regarding input and output data formats. Third, the predictive accuracy of the nomogram can still be improved, which may imply the need for the inclusion of additional factors such as physical examination results and survey data. The prediction accuracy could perhaps be improved in further studies with a wider population coverage, larger sample size, and more variables.

### Conclusions

To promptly and accurately identify children and adolescents at high risk of PH with maximum efficiency and at a low cost, aiming to minimize the potential harm to this population, we develop and validate a straightforward and dependable nomogram. The nomogram uses conventional EHR data to predict risk with strong accuracy, discrimination, and clinical utility in both training and validation cohorts, signifying its robust performance in practical applications. This visual model and accompanying website will assist patients and physicians in forecasting the PH risk and enhance clinical management in a timely manner.

## Appendix

Multimedia Appendix 1 Tables and figures for the study.

Multimedia Appendix 2 Table of baseline characteristics of individuals in the training cohort and validation cohort.

## Abbreviations

AH: arterial hypertension
AUC: area under the receiver operating characteristic curve
C-index: concordance index
DCA: decision curve analysis
EHR: electronic health record
FBG: fasting blood glucose
IBIL: indirect bilirubin
ICD-10-CM: International Classification of Diseases, 10th Revision, Clinical Modification Lasso: least absolute shrinkage and selection operator
LDL-C: low-density lipoprotein cholesterol
LYC: lymphocyte count
LYP: lymphocyte percentage
MCV: mean corpuscular volume
minMSE: minimum mean squared error
MONOC: monocyte count
NEUTP: neutrophil percentage
PDW: platelet distribution
PH: primary hypertension
OR: odds ratio
SH: secondary hypertension
TC: total cholesterol
UA: uric acid
