# Supplementary material for: Development and Validation of a Nomogram-Based Model to Predict Primary Hypertension Within the Next Year in Children and Adolescents: Retrospective Cohort Study
Source: J Med Internet Res. 2024 Dec 30;26:e58686. doi: 10.2196/58686 (PMC11730233; doi:10.2196/58686)
Supplement: Multimedia Appendix 1 [file jmir_v26i1e58686_app1.docx]

**Table S1.** Variables included in the pre-processing, training, and external validation stages.

**Table S2.** Baseline characteristics of individuals in training cohort and external validation cohort.

**Table S3.** Comparison of baseline characteristics between PH patients and non-PH patients.

**Table S4.** Comparison of baseline characteristics between subjects aged 11 years or younger and subjects aged over 11 years.

**Table S5.** Comparison of baseline characteristics between male and female.

**Figure S1.** The distribution of primary hypertension in boys and girls aged 6-18 years (n=521).

**Table S1.** Variables included in the pre-processing, training, and external validation stages.

| Variable name | Available in > 80% of EHR | Included in LASSO model training | Included in Logistic model training | Included in external validation |
| --- | --- | --- | --- | --- |
|  |  |  |  |  |
| Family history of hypertension | ✔ | ✔ | ✔ | ✔ |
| Family history of diabetes | ✔ | ✔ |  |  |
| Breastfeeding | ✔ | ✔ | ✔ | ✔ |
| Gender | ✔ | ✔ | ✔ | ✔ |
| AGE | ✔ | ✔ | ✔ | ✔ |
| White/bulb ratio | ✔ | ✔ |  |  |
| Albumin | ✔ | ✔ |  |  |
| Creatine kinase | ✔ | ✔ |  |  |
| Calcium | ✔ | ✔ |  |  |
| Creatinine | ✔ | ✔ |  |  |
| Direct bilirubin | ✔ | ✔ |  |  |
| Fasting blood glucose | ✔ | ✔ | ✔ | ✔ |
| Globulin | ✔ | ✔ |  |  |
| Hematocrit | ✔ | ✔ |  |  |
| High-density lipoprotein cholesterol | ✔ | ✔ |  |  |
| Hemoglobin | ✔ | ✔ |  |  |
| Indirect bilirubin | ✔ | ✔ |  |  |
| Kalium | ✔ | ✔ |  |  |
| Lactate dehydrogenase | ✔ | ✔ |  |  |
| Low-density lipoprotein cholesterol | ✔ | ✔ | ✔ | ✔ |
| Lipoprotein (a) | ✔ | ✔ |  |  |
| Lymphocyte count | ✔ | ✔ |  |  |
| Lymphocyte percentage | ✔ | ✔ |  |  |
| Mean corpuscular hemoglobin | ✔ |  |  |  |
| Mean cell hemoglobin concentration | ✔ | ✔ |  |  |
| Mean corpuscular volume | ✔ | ✔ |  |  |
| Monocyte count | ✔ | ✔ |  |  |
| Monocyte percentage | ✔ | ✔ |  |  |
| Mean platelet volume | ✔ | ✔ |  |  |
| Neutrophil count | ✔ | ✔ |  |  |
| Neutrophil percentage | ✔ | ✔ |  |  |
| Natrium | ✔ | ✔ |  |  |
| Platelet crit | ✔ | ✔ |  |  |
| Platelet distribution | ✔ | ✔ |  |  |
| Platelet count | ✔ | ✔ |  |  |
| Red blood cell count | ✔ |  |  |  |
| RDWCV^a^ | ✔ | ✔ |  |  |
| Total bilirubin | ✔ | ✔ |  |  |
| Total cholesterol | ✔ | ✔ |  |  |
| Triglycerides | ✔ |  |  |  |
| Total protein | ✔ |  |  |  |
| Uric acid | ✔ | ✔ | ✔ | ✔ |
| White blood cell count | ✔ |  |  |  |
| urea | ✔ |  |  |  |
| HBDH^b^ |  |  |  |  |
| adenosine deaminase |  |  |  |  |
| γ-glutamyl transpeptidase |  |  |  |  |
| alkaline phosphatase |  |  |  |  |
| alanine aminotransferase |  |  |  |  |
| aspartate aminotransferase |  |  |  |  |
| retinol-binding protein |  |  |  |  |
| phosphorus |  |  |  |  |
| magnesium |  |  |  |  |
| chlorine |  |  |  |  |
| basophil count |  |  |  |  |
| basophil percentage |  |  |  |  |
| eosinophil count |  |  |  |  |
| eosinophil percentage |  |  |  |  |

^a^RDWCV: red blood cell distribution width-coefficient of variation.

^b^HBDH: α-hydroxybutyrate dehydrogenase.

**Table S2.** Baseline characteristics of individuals in training cohort and external validation cohort.

| Characteristics | Training cohort (N = 3,938) | External validation cohort (N = 576) | *P* values |
| --- | --- | --- | --- |
|  |  |  |  |
| Diagnosis of primary hypertension |  |  |  |
| No | 3,553 (90.2) | 523 (90.8) | .71 |
| Yes | 385 (9.8) | 53 (9.2) |  |
| Family history of hypertension |  |  |  |
| No | 3,882 (98.6) | 554 (96.2) | <.001 |
| Yes | 56 (1.4) | 22 (3.8) |  |
| Family history of diabetes |  |  |  |
| No | 3,923 (99.6) | 571 (99.1) | .17 |
| Yes | 15 (0.4) | 5 (0.9) |  |
| Breastfeeding |  |  |  |
| No | 2,171 (55.1) | 496 (86.1) | <.001 |
| Yes | 1,767 (44.9) | 80 (13.9) |  |
| Gender |  |  |  |
| Female | 2,186 (55.5) | 343 (59.5) | .09 |
| Male | 1,752 (44.5) | 233 (40.5) |  |
| AGE (years), median (IQR) | 14.00 [11.00, 16.00] | 13.50 [10.00, 15.00] | .3 |
| White/bulb ratio (%), median (IQR) | 1.61 [1.57, 1.67] | 1.59 [1.47, 1.72] | <.001 |
| Albumin (g/L), median (IQR) | 42.72 [42.01, 43.07] | 42.73 [37.70, 44.63] | .05 |
| Creatine kinase (U/L), median (IQR) | 121.00 [106.08, 158.86] | 104.68 [75.00, 141.25] | <.001 |
| Calcium (mmol/L), median (IQR) | 2.35 [2.23, 2.44] | 2.37 [2.29, 2.44] | 0.006 |
| Creatinine (umol/L), median (IQR) | 46.38 [38.51, 62.29] | 56.00 [38.69, 67.48] | <.001 |
| Direct bilirubin (μmol/L), median (IQR) | 3.88 [3.53, 4.20] | 3.88 [2.80, 4.47] | .1 |
| Fasting blood glucose (mmol/L), median (IQR) | 4.92 [4.85, 4.99] | 4.95 [4.84, 5.15] | .2 |
| Globulin (g/L), median (IQR) | 27.63 [26.53, 27.99] | 27.60 [23.58, 28.82] | .1 |
| Hematocrit (%), median (IQR) | 0.42 (0.03) | 37.55 [0.44, 42.32] | <.001 |
| High-density lipoprotein cholesterol (mmol/L), median (IQR) | 1.23 [1.14, 1.32] | 1.24 [1.12, 1.38] | .08 |
| Hemoglobin (g/L), median (IQR) | 135.37 [129.00, 144.78] | 136.00 [126.00, 152.00] | .03 |
| Indirect bilirubin (μmol/L), median (IQR) | 7.19 (1.05) | 7.40 [6.70, 8.34] | <.001 |
| Kalium (mmol/L), median (IQR) | 4.18 [4.11, 4.26] | 4.19 [4.05, 4.43] | .04 |
| Lactate dehydrogenase (U/L), median (IQR) | 224.72 [202.60, 245.99] | 208.83 [175.30, 262.24] | <.001 |
| Low-density lipoprotein cholesterol (mmol/L), median (IQR) | 2.65 [2.46, 2.72] | 2.51 [2.35, 2.74] | .09 |
| Lipoprotein (a) (mg/L), median (IQR) | 183.70 [152.89, 193.20] | 180.57 [128.39, 180.72] | <.001 |
| Lymphocyte count (10^9^/L), median (IQR) | 2.16 (0.85) | 2.33 [1.74, 3.11] | <.001 |
| Lymphocyte percentage (%), median (IQR) | 29.41 (11.10) | 32.08 [24.58, 40.25] | <.001 |
| Mean corpuscular hemoglobin (pg), median (IQR) | 28.65 [27.80, 29.40] | 29.60 [28.10, 30.96] | <.001 |
| Mean cell hemoglobin concentration (g/L), median (IQR) | 328.61 [316.00, 339.00] | 334.67 [328.32, 341.00] | <.001 |
| Mean corpuscular volume (fL), median (IQR) | 87.58 (4.12) | 87.80 [84.72, 91.96] | .09 |
| Monocyte count (10^9^/L), median (IQR) | 0.60 [0.47, 0.68] | 0.58 [0.43, 0.80] | .2 |
| Monocyte percentage (%), median (IQR) | 7.80 [6.47, 10.00] | 7.55 [6.20, 9.36] | 0.002 |
| Mean platelet volume (fL), median (IQR) | 10.20 [9.70, 10.70] | 9.78 [9.20, 10.40] | <.001 |
| Neutrophil count (10^9^/L), median (IQR) | 4.71 [3.27, 6.09] | 4.41 [3.33, 6.02] | .6 |
| Neutrophil percentage (%), median (IQR) | 59.95 (11.89) | 58.05 [48.00, 66.01] | <.001 |
| Natrium (mmol/L), median (IQR) | 138.35 [137.62, 139.26] | 138.40 [136.80, 139.70] | .03 |
| Platelet crit (%), median (IQR) | 0.23 [0.20, 0.26] | 0.25 [0.22, 0.30] | <.001 |
| Platelet distribution (fL), median (IQR) | 11.65 (1.81) | 14.00 [11.27, 15.90] | <.001 |
| Platelet count (109/L), median (IQR) | 231.00 [194.00, 264.00] | 255.50 [216.75, 310.56] | <.001 |
| Red blood cell count (10^12^/L), median (IQR) | 4.75 (0.37) | 4.66 [4.37, 4.99] | <.001 |
| RDWCV^a^ (%), median (IQR) | 12.34 [11.93, 12.73] | 12.60 [12.00, 13.89] | <.001 |
| Total bilirubin (μmol/L), median (IQR) | 11.24 [10.73, 11.61] | 11.45 [8.91, 16.70] | <.001 |
| Total cholesterol (mmol/L), median (IQR) | 4.11 [3.92, 4.25] | 4.15 [3.81, 4.44] | .04 |
| Triglycerides (mmol/L), median (IQR) | 1.10 [0.88, 1.27] | 1.03 [0.77, 1.23] | .05 |
| Total protein (g/L), median (IQR) | 70.27 [69.31, 71.04] | 70.03 [63.77, 73.22] | <.001 |
| Uric acid (umol/L), median (IQR) | 340.00 [293.83, 369.55] | 343.28 [296.87, 409.21] | .07 |
| White blood cell count (10^9^/L), median (IQR) | 7.74 [6.14, 9.22] | 8.03 [6.63, 9.88] | <.001 |
| urea (mmol/L), median (IQR) | 4.37 [4.06, 4.80] | 4.50 [4.17, 4.83] | .06 |

^a^RDWCV: red blood cell distribution width-coefficient of variation.

**Table S3.** Comparison of baseline characteristics between PH^a^ patients and non-PH^a^ patients.

| Characteristics | Training cohort | | Validation cohort | |
| --- | --- | --- | --- | --- |
|  | Patients with PH | Patients without PH | Patients with PH | Patients without PH |
|  | n = 385 | n = 3553 | n = 136 | n = 1133 |
|  |  |  |  |  |
| Family history of hypertension |  |  |  |  |
| No | 343 (89.1) | 3539 (99.6) | 123 (90.4) | 1131 (99.8) |
| Yes | 42 (10.9) | 14 (0.4) | 13 (9.6) | 2 (0.2) |
| Family history of diabetes |  |  |  |  |
| No | 381 (99.0) | 3542 (99.7) | 135 (99.3) | 1128 (99.6) |
| Yes | 4 (1.0) | 11 (0.3) | 1 (0.7) | 5 (0.4) |
| Breastfeeding |  |  |  |  |
| No | 376 (97.7) | 1795 (50.5) | 134 (98.5) | 839 (74.1) |
| Yes | 9 (2.3) | 1758 (49.5) | 2 (1.5) | 294 (25.9) |
| Gender |  |  |  |  |
| Female | 86 (22.3) | 2100 (59.1) | 21 (15.4) | 671 (59.2) |
| Male | 299 (77.7) | 1453 (40.9) | 115 (84.6) | 462 (40.8) |
| AGE | 16.00 [14.00, 17.00] | 12.00 [9.00, 16.00] | 16.00 [15.00, 17.00] | 15.00 [11.00, 17.00] |
| White/bulb ratio (%) | 1.69 [1.61, 1.75] | 1.61 [1.56, 1.66] | 1.62 [1.50, 1.70] | 1.58 [1.54, 1.61] |
| Albumin (g/L) | 45.73 [45.00, 46.45] | 42.60 [41.95, 42.84] | 45.36 [44.52, 46.73] | 44.34 [43.72, 44.82] |
| Creatine kinase (U/L) | 115.26 [102.96, 123.92] | 124.03 [107.00, 161.91] | 102.64 [91.50, 112.46] | 98.94 [85.07, 109.00] |
| Calcium (mmol/L) | 2.42 [2.40, 2.45] | 2.34 [2.22, 2.44] | 2.42 [2.38, 2.47] | 2.36 [2.30, 2.43] |
| Creatinine (umol/L) | 66.62 [60.80, 68.63] | 43.90 [38.32, 59.18] | 66.85 [61.68, 71.00] | 57.70 [47.00, 69.30] |
| Direct bilirubin (μmol/L) | 4.30 [3.79, 4.46] | 3.85 [3.52, 4.14] | 4.55 [3.88, 4.67] | 4.15 [3.50, 4.90] |
| Fasting blood glucose (mmol/L) | 5.00 [4.82, 5.13] | 4.92 [4.85, 4.98] | 4.75 [4.68, 4.96] | 5.10 [4.96, 5.16] |
| Globulin (g/L) | 27.37 [26.53, 28.20] | 27.66 [26.53, 27.99] | 27.52 [26.35, 29.22] | 28.69 [27.50, 29.03] |
| Hematocrit (%) | 0.43 [0.42, 0.44] | 0.41 [0.40, 0.43] | 0.43 [0.43, 0.45] | 0.41 [0.39, 0.43] |
| High-density lipoprotein cholesterol (mmol/L) | 1.17 [1.12, 1.22] | 1.24 [1.14, 1.33] | 1.10 [1.05, 1.20] | 1.27 [1.14, 1.40] |
| Hemoglobin (g/L) | 143.94 [141.00, 146.84] | 135.00 [128.00, 140.00] | 145.41 [141.37, 152.00] | 135.97 [129.00, 142.86] |
| Indirect bilirubin (μmol/L) | 7.97 [7.10, 8.34] | 7.35 [7.08, 7.52] | 7.69 [6.68, 8.20] | 7.20 [6.30, 8.00] |
| Kalium (mmol/L) | 4.24 [4.19, 4.28] | 4.17 [4.11, 4.25] | 4.20 [4.15, 4.30] | 4.13 [4.06, 4.20] |
| Lactate dehydrogenase (U/L) | 201.10 [192.00, 211.07] | 227.71 [205.32, 247.47] | 212.88 [189.00, 217.28] | 199.00 [174.03, 224.01] |
| Low-density lipoprotein cholesterol (mmol/L) | 2.54 [2.43, 2.59] | 2.44 [2.36, 2.51] | 2.58 [2.40, 2.67] | 2.68 [2.56, 2.80] |
| Lipoprotein (a) (mg/L) | 151.99 [109.00, 173.67] | 187.34 [158.46, 195.05] | 121.66 [70.50, 134.87] | 180.56 [109.00, 180.67] |
| Lymphocyte count (10^9^/L) | 2.14 [1.93, 2.23] | 2.08 [1.57, 2.64] | 2.14 [2.09, 2.33] | 2.11 [1.65, 2.56] |
| Lymphocyte percentage (%) | 29.06 [27.58, 31.00] | 29.10 [21.10, 37.00] | 28.97 [27.30, 30.69] | 29.67 [24.48, 36.20] |
| Mean corpuscular hemoglobin (pg) | 28.91 [28.70, 29.17] | 28.60 [27.80, 29.40] | 29.05 [28.78, 29.41] | 28.95 [28.30, 29.75] |
| Mean cell hemoglobin concentration (g/L) | 334.65 [333.06, 339.00] | 328.01 [314.50, 339.00] | 334.60 [330.62, 335.80] | 330.30 [326.00, 335.00] |
| Mean corpuscular volume (fL) | 86.46 [85.90, 86.90] | 87.53 [85.00, 90.40] | 86.77 [86.23, 87.82] | 87.57 [85.67, 89.67] |
| Monocyte count (10^9^/L) | 0.56 [0.48, 0.58] | 0.62 [0.47, 0.80] | 0.58 [0.53, 0.60] | 0.57 [0.45, 0.65] |
| Monocyte percentage (%) | 7.23 [6.66, 7.49] | 8.10 [6.40, 10.20] | 7.34 [6.73, 7.72] | 7.68 [6.40, 8.90] |
| Mean platelet volume (fL) | 10.06 [9.68, 10.50] | 10.20 [9.70, 10.75] | 10.66 [10.00, 10.90] | 9.81 [9.40, 10.30] |
| Neutrophil count (10^9^/L) | 5.03 [4.47, 5.19] | 4.62 [3.16, 6.18] | 4.94 [4.72, 5.62] | 4.57 [3.42, 5.27] |
| Neutrophil percentage (%) | 61.31 [59.24, 62.81] | 60.11 [51.60, 68.20] | 60.85 [58.08, 63.05] | 59.55 [52.70, 65.27] |
| Natrium (mmol/L) | 140.00 [139.70, 140.08] | 138.19 [137.58, 139.03] | 139.87 [139.45, 140.05] | 138.80 [138.00, 139.67] |
| Platelet crit (%) | 0.24 [0.22, 0.25] | 0.23 [0.20, 0.27] | 0.25 [0.24, 0.27] | 0.25 [0.22, 0.28] |
| Platelet distribution (fL) | 13.40 [12.72, 13.73] | 11.25 [10.20, 12.50] | 12.87 [12.16, 13.21] | 11.20 [10.10, 12.16] |
| Platelet count (109/L) | 233.54 [217.00, 251.50] | 230.21 [191.00, 266.00] | 257.36 [237.34, 270.88] | 257.67 [224.00, 286.33] |
| Red blood cell count (10^12^/L) | 4.99 [4.90, 5.07] | 4.73 [4.50, 4.94] | 4.99 [4.90, 5.19] | 4.71 [4.46, 4.95] |
| RDWCV^b^ (%) | 12.64 [12.55, 12.73] | 12.30 [11.90, 12.74] | 12.73 [12.50, 12.78] | 12.48 [12.10, 12.80] |
| Total bilirubin (μmol/L) | 12.53 [11.13, 12.93] | 11.21 [10.72, 11.55] | 12.35 [11.00, 12.71] | 11.44 [10.03, 12.87] |
| Total cholesterol (mmol/L) | 4.14 [3.94, 4.26] | 4.10 [3.92, 4.25] | 4.19 [3.79, 4.42] | 4.38 [4.16, 4.56] |
| Triglycerides (mmol/L) | 1.15 [0.95, 1.22] | 0.95 [0.77, 1.15] | 1.24 [1.01, 1.28] | 1.14 [0.89, 1.28] |
| Total protein (g/L) | 73.13 [72.39, 73.94] | 70.19 [69.31, 70.81] | 72.90 [71.98, 74.80] | 73.13 [72.05, 73.50] |
| Uric acid (umol/L) | 380.93 [354.29, 403.49] | 303.57 [271.54, 357.00] | 416.35 [379.38, 432.61] | 330.04 [296.43, 377.33] |
| White blood cell count (10^9^/L) | 7.99 [7.40, 8.10] | 7.65 [6.08, 9.34] | 7.97 [7.70, 8.74] | 7.60 [6.23, 8.43] |
| urea (mmol/L) | 4.61 [4.39, 4.80] | 4.36 [4.03, 4.80] | 4.86 [4.40, 4.98] | 4.47 [4.13, 4.82] |

^a^PH: primary hypertension.

^b^RDWCV: red blood cell distribution width-coefficient of variation.

We first categorized the subjects into two subgroups based on their PH status both in the training cohort and validation cohort. The characteristics of subjects in subgroups are shown in Table S3 in Multimedia Appendix 1. Family history of hypertension was recorded for 10.9% (42 subjects) of subjects with PH and 0.4% (14 subjects) of subjects without PH in the training cohort, we saw similar results in the validation cohort. In the breastfeeding status, we found that people with no history of breastfeeding accounted for 97.7% (376 subjects) of PH patients in the training cohort and 98.5% (134 subjects) in the validation cohort. Same as the result in the distribution of PH (Figure S1 in Multimedia Appendix 1), in the training cohort, males constituted a substantial majority among PH patients, comprising 77.7% (299 subjects), in stark contrast to females who accounted for only 22.3% (86 subjects). Additionally, individuals without PH were generally younger than their counterparts with PH, and the UA levels of PH patients were significantly higher than those without PH in the training cohort. These trends were consistently observed in the validation cohort.

**Table S4.** Comparison of baseline characteristics between subjects aged 11 years or younger and subjects aged over 11 years.

| Characteristics | Training cohort | | Validation cohort | |
| --- | --- | --- | --- | --- |
|  | Age ≤ 11 | Age > 11 | Age ≤ 11 | Age > 11 |
|  | n = 1795 | n = 2143 | n = 325 | n = 944 |
|  |  |  |  |  |
| Diagnosis of PH |  |  |  |  |
| No | 1770 (98.6) | 1783 (83.2) | 322 (99.1) | 811 (85.9) |
| Yes | 25 (1.4) | 360 (16.8) | 3 (0.9) | 133 (14.1) |
| Family history of hypertension |  |  |  |  |
| No | 1790 (99.7) | 2092 (97.6) | 325 (100.0) | 929 (98.4) |
| Yes | 5 (0.3) | 51 (2.4) | 0 (0.0) | 15 (1.6) |
| Family history of diabetes |  |  |  |  |
| No | 1793 (99.9) | 2130 (99.4) | 325 (100.0) | 938 (99.4) |
| Yes | 2 (0.1) | 13 (0.6) | 0 (0.0) | 6 (0.6) |
| Breastfeeding |  |  |  |  |
| No | 491 (27.4) | 1680 (78.4) | 124 (38.2) | 849 (89.9) |
| Yes | 1304 (72.6) | 463 (21.6) | 201 (61.8) | 95 (10.1) |
| Gender |  |  |  |  |
| Female | 1160 (64.6) | 1026 (47.9) | 216 (66.5) | 476 (50.4) |
| Male | 635 (35.4) | 1117 (52.1) | 109 (33.5) | 468 (49.6) |
| White/bulb ratio (%) | 1.64 [1.60, 1.68] | 1.59 [1.54, 1.65] | 1.60 [1.54, 1.66] | 1.58 [1.54, 1.61] |
| Albumin (g/L) | 42.42 [41.78, 42.77] | 42.80 [42.27, 44.24] | 43.77 [42.90, 44.45] | 44.50 [44.10, 45.50] |
| Creatine kinase (U/L) | 124.84 [118.94, 169.12] | 119.97 [91.85, 149.45] | 102.61 [93.64, 115.76] | 97.18 [83.39, 107.57] |
| Calcium (mmol/L) | 2.29 [2.16, 2.42] | 2.39 [2.30, 2.46] | 2.34 [2.29, 2.42] | 2.38 [2.31, 2.44] |
| Creatinine (umol/L) | 38.55 [32.42, 40.76] | 60.35 [49.72, 67.90] | 44.00 [39.30, 48.70] | 65.40 [56.25, 72.14] |
| Direct bilirubin (μmol/L) | 3.66 [3.40, 3.90] | 4.10 [3.79, 4.36] | 3.88 [2.90, 4.20] | 4.41 [3.80, 5.00] |
| Fasting blood glucose (mmol/L) | 4.96 [4.91, 5.03] | 4.88 [4.82, 4.95] | 5.08 [4.94, 5.20] | 5.09 [4.83, 5.15] |
| Globulin (g/L) | 27.40 [26.53, 27.64] | 27.91 [27.46, 28.16] | 28.00 [26.88, 28.70] | 28.75 [27.82, 29.14] |
| Hematocrit (%) | 0.41 [0.39, 0.43] | 0.42 [0.40, 0.44] | 0.40 [0.39, 0.41] | 0.42 [0.40, 0.44] |
| High-density lipoprotein cholesterol (mmol/L) | 1.26 [1.17, 1.35] | 1.20 [1.11, 1.30] | 1.35 [1.17, 1.44] | 1.23 [1.11, 1.37] |
| Hemoglobin (g/L) | 131.50 [126.00, 136.00] | 138.00 [132.02, 146.33] | 131.00 [127.00, 136.60] | 139.00 [131.00, 147.00] |
| Indirect bilirubin (μmol/L) | 7.38 [7.09, 7.56] | 7.35 [7.08, 7.56] | 6.60 [5.43, 7.05] | 7.51 [6.72, 8.15] |
| Kalium (mmol/L) | 4.17 [4.12, 4.25] | 4.19 [4.10, 4.26] | 4.13 [4.06, 4.20] | 4.14 [4.06, 4.21] |
| Lactate dehydrogenase (U/L) | 243.24 [230.45, 256.81] | 206.51 [194.33, 222.00] | 224.10 [215.00, 246.00] | 189.98 [169.00, 212.61] |
| Low-density lipoprotein cholesterol (mmol/L) | 2.42 [2.33, 2.47] | 2.48 [2.40, 2.54] | 2.65 [2.45, 2.81] | 2.68 [2.57, 2.80] |
| Lipoprotein (a) (mg/L) | 189.39 [165.73, 189.48] | 176.00 [143.00, 196.37] | 166.68 [65.50, 180.67] | 180.55 [115.95, 180.66] |
| Lymphocyte count (10^9^/L) | 2.16 [1.55, 2.79] | 2.07 [1.63, 2.45] | 2.28 [1.99, 2.78] | 2.11 [1.63, 2.42] |
| Lymphocyte percentage (%) | 30.00 [21.50, 39.80] | 28.80 [21.40, 34.10] | 33.70 [28.73, 40.75] | 29.39 [23.40, 34.23] |
| Mean corpuscular hemoglobin (pg) | 28.15 [27.40, 28.70] | 29.00 [28.50, 29.93] | 28.40 [27.70, 28.90] | 29.05 [28.60, 29.90] |
| Mean cell hemoglobin concentration (g/L) | 319.51 [309.00, 331.00] | 334.05 [327.15, 342.00] | 330.35 [326.00, 333.50] | 330.52 [326.50, 335.27] |
| Mean corpuscular volume (fL) | 87.60 [84.60, 90.50] | 87.23 [85.70, 89.70] | 86.14 [84.10, 87.50] | 87.70 [86.43, 90.23] |
| Monocyte count (10^9^/L) | 0.64 [0.47, 0.83] | 0.58 [0.47, 0.74] | 0.53 [0.43, 0.63] | 0.58 [0.47, 0.66] |
| Monocyte percentage (%) | 8.59 [6.66, 11.10] | 7.40 [6.30, 9.10] | 7.30 [6.10, 8.20] | 7.70 [6.60, 9.00] |
| Mean platelet volume (fL) | 10.10 [9.64, 10.70] | 10.20 [9.70, 10.75] | 9.80 [9.40, 10.22] | 9.90 [9.47, 10.50] |
| Neutrophil count (10^9^/L) | 4.36 [2.92, 5.93] | 4.96 [3.51, 6.24] | 4.19 [3.19, 4.80] | 4.73 [3.60, 5.52] |
| Neutrophil percentage (%) | 58.57 [48.77, 67.10] | 61.26 [55.10, 68.30] | 55.90 [49.00, 60.43] | 59.89 [55.00, 66.50] |
| Natrium (mmol/L) | 137.64 [137.46, 138.07] | 139.08 [138.46, 139.76] | 138.00 [137.38, 138.54] | 139.21 [138.50, 139.99] |
| Platelet crit (%) | 0.22 [0.19, 0.26] | 0.24 [0.21, 0.27] | 0.26 [0.24, 0.29] | 0.25 [0.22, 0.28] |
| Platelet distribution (fL) | 10.84 [9.90, 12.01] | 12.00 [10.70, 13.35] | 11.10 [10.10, 11.91] | 11.42 [10.30, 12.84] |
| Platelet count (109/L) | 221.00 [182.00, 259.00] | 235.92 [204.06, 267.00] | 265.20 [244.56, 297.79] | 255.74 [220.44, 279.50] |
| Red blood cell count (10^12^/L) | 4.70 [4.48, 4.88] | 4.78 [4.56, 5.03] | 4.61 [4.48, 4.83] | 4.80 [4.48, 5.03] |
| RDWCV^a^ (%) | 12.20 [11.85, 12.60] | 12.50 [12.00, 12.83] | 12.40 [12.10, 12.60] | 12.56 [12.10, 12.85] |
| Total bilirubin (μmol/L) | 11.00 [10.64, 11.31] | 11.48 [11.01, 11.91] | 10.49 [8.40, 10.92] | 12.06 [10.90, 13.15] |
| Total cholesterol (mmol/L) | 4.03 [3.88, 4.16] | 4.18 [3.99, 4.31] | 4.34 [4.05, 4.52] | 4.38 [4.18, 4.56] |
| Triglycerides (mmol/L) | 0.85 [0.71, 1.01] | 1.09 [0.89, 1.24] | 0.93 [0.83, 1.10] | 1.20 [1.03, 1.32] |
| Total protein (g/L) | 69.71 [69.31, 70.17] | 70.81 [70.30, 72.40] | 72.00 [69.80, 73.02] | 73.23 [72.84, 73.80] |
| Uric acid (umol/L) | 278.56 [263.21, 307.95] | 353.70 [306.33, 395.95] | 296.82 [270.00, 318.46] | 357.84 [314.60, 403.24] |
| White blood cell count (10^9^/L) | 7.57 [5.96, 9.09] | 7.88 [6.32, 9.31] | 7.55 [6.31, 8.06] | 7.64 [6.32, 8.62] |
| urea (mmol/L) | 4.35 [4.01, 4.60] | 4.52 [4.08, 4.92] | 4.28 [4.03, 4.62] | 4.59 [4.22, 4.90] |

^a^RDWCV: red blood cell distribution width-coefficient of variation.

In Figure S1 in Multimedia Appendix 1, we observed a significant increase in the risk of developing PH in children and adolescents after the age of 11, so we divided the subjects into two subgroups using the age of 11 as the cutoff point. The baseline characteristics of two subgroups are displayed in Table S4 in Multimedia Appendix 1. In the training cohort, diagnoses with PH were recorded for 1.4% (25 subjects) of subjects aged 11 years or younger and 16.8% (360 subjects) of subjects aged over 11 years, the results in the validation cohort are approximately similar. It is worth noting that the UA levels of subjects aged 11 years or younger were all obviously lower than those aged over 11 years both in training and validation cohorts.

**Table S5.** Comparison of baseline characteristics between male and female.

| Characteristics | Training cohort | | Validation cohort | |
| --- | --- | --- | --- | --- |
|  | Female | Male | Female | Male |
|  | n=2186 | n=1752 | n=692 | n=577 |
| Diagnosis of PH |  |  |  |  |
| No | 2100 (96.1) | 1453 (82.9) | 671 (97.0) | 462 (80.1) |
| Yes | 86 (3.9) | 299 (17.1) | 21 (3.0) | 115 (19.9) |
| Family history of hypertension |  |  |  |  |
| No | 2171 (99.3) | 1711 (97.7) | 690 (99.7) | 564 (97.7) |
| Yes | 15 (0.7) | 41 (2.3) | 2 (0.3) | 13 (2.3) |
| Family history of diabetes |  |  |  |  |
| No | 2179 (99.7) | 1744 (99.5) | 690 (99.7) | 573 (99.3) |
| Yes | 7 (0.3) | 8 (0.5) | 2 (0.3) | 4 (0.7) |
| Breastfeeding |  |  |  |  |
| No | 1172 (53.6) | 999 (57.0) | 521 (75.3) | 452 (78.3) |
| Yes | 1014 (46.4) | 753 (43.0) | 171 (24.7) | 125 (21.7) |
| AGE (years) | 11.00 [9.00, 15.00] | 14.00 [10.00, 16.00] | 14.00 [11.00, 17.00] | 16.00 [13.00, 17.00] |
| White/bulb ratio (%) | 1.62 [1.57, 1.67] | 1.61 [1.56, 1.67] | 1.58 [1.54, 1.62] | 1.59 [1.55, 1.63] |
| Albumin (g/L) | 42.75 [42.09, 42.77] | 42.69 [41.94, 44.25] | 44.19 [43.24, 44.59] | 44.68 [44.23, 46.00] |
| Creatine kinase (U/L) | 119.19 [102.16, 147.86] | 136.27 [109.27, 168.05] | 93.67 [77.00, 102.70] | 105.00 [95.20, 115.68] |
| Calcium (mmol/L) | 2.34 [2.23, 2.43] | 2.38 [2.24, 2.45] | 2.35 [2.29, 2.41] | 2.41 [2.33, 2.46] |
| Creatinine (umol/L) | 38.93 [37.10, 51.20] | 59.78 [44.33, 68.50] | 53.39 [45.70, 62.44] | 68.30 [57.57, 75.20] |
| Direct bilirubin (μmol/L) | 3.73 [3.42, 4.04] | 4.06 [3.74, 4.37] | 3.98 [3.44, 4.68] | 4.50 [3.70, 5.03] |
| Fasting blood glucose (mmol/L) | 4.94 [4.88, 5.03] | 4.89 [4.83, 4.95] | 5.08 [4.93, 5.15] | 5.10 [4.81, 5.17] |
| Globulin (g/L) | 27.61 [26.53, 28.00] | 27.65 [27.31, 27.98] | 28.70 [27.01, 29.03] | 28.63 [27.50, 29.04] |
| Hematocrit (%) | 0.41 [0.39, 0.42] | 0.43 [0.41, 0.45] | 0.40 [0.38, 0.41] | 0.43 [0.42, 0.46] |
| High-density lipoprotein cholesterol (mmol/L) | 1.28 [1.20, 1.36] | 1.18 [1.09, 1.25] | 1.34 [1.20, 1.43] | 1.17 [1.07, 1.28] |
| Hemoglobin (g/L) | 132.00 [127.00, 137.00] | 140.00 [133.50, 148.24] | 131.00 [126.00, 136.50] | 145.00 [138.17, 152.50] |
| Indirect bilirubin (μmol/L) | 7.38 [6.95, 7.57] | 7.35 [7.17, 7.52] | 7.04 [6.31, 7.80] | 7.55 [6.33, 8.16] |
| Kalium (mmol/L) | 4.16 [4.11, 4.23] | 4.21 [4.13, 4.29] | 4.11 [4.05, 4.19] | 4.17 [4.09, 4.24] |
| Lactate dehydrogenase (U/L) | 229.51 [207.04, 251.33] | 216.79 [198.91, 239.01] | 196.69 [170.00, 223.94] | 206.30 [182.33, 228.00] |
| Low-density lipoprotein cholesterol (mmol/L) | 2.43 [2.32, 2.50] | 2.47 [2.40, 2.54] | 2.66 [2.55, 2.76] | 2.70 [2.54, 2.82] |
| Lipoprotein (a) (mg/L) | 189.40 [165.90, 194.26] | 171.41 [142.26, 192.43] | 180.60 [121.55, 180.70] | 180.41 [71.00, 180.60] |
| Lymphocyte count (10^9^/L) | 2.10 [1.56, 2.65] | 2.13 [1.64, 2.55] | 2.11 [1.65, 2.53] | 2.13 [1.74, 2.49] |
| Lymphocyte percentage (%) | 29.40 [21.33, 36.99] | 28.93 [21.60, 35.59] | 29.80 [24.90, 36.90] | 29.37 [24.83, 33.50] |
| Mean corpuscular hemoglobin (pg) | 28.60 [27.80, 29.40] | 28.70 [27.90, 29.40] | 28.94 [28.26, 29.75] | 28.97 [28.40, 29.70] |
| Mean cell hemoglobin concentration (g/L) | 326.50 [313.00, 336.00] | 333.00 [322.00, 342.00] | 329.81 [325.00, 333.00] | 332.00 [329.00, 337.00] |
| Mean corpuscular volume (fL) | 88.10 [85.60, 91.04] | 86.87 [84.80, 88.70] | 87.67 [85.87, 90.20] | 87.32 [85.40, 88.76] |
| Monocyte count (10^9^/L) | 0.60 [0.45, 0.76] | 0.61 [0.49, 0.80] | 0.54 [0.43, 0.63] | 0.59 [0.49, 0.70] |
| Monocyte percentage (%) | 7.83 [6.25, 10.10] | 7.80 [6.70, 9.89] | 7.40 [6.20, 8.63] | 7.70 [6.75, 9.03] |
| Mean platelet volume (fL) | 10.20 [9.65, 10.70] | 10.19 [9.70, 10.70] | 9.89 [9.40, 10.30] | 9.89 [9.46, 10.60] |
| Neutrophil count (10^9^/L) | 4.63 [3.13, 6.03] | 4.87 [3.38, 6.15] | 4.44 [3.27, 5.39] | 4.73 [3.69, 5.27] |
| Neutrophil percentage (%) | 60.40 [52.00, 68.20] | 60.50 [52.40, 67.36] | 59.68 [52.69, 65.70] | 59.70 [54.80, 64.40] |
| Natrium (mmol/L) | 137.97 [137.54, 138.89] | 138.82 [138.00, 139.67] | 138.54 [137.95, 139.16] | 139.60 [138.55, 140.11] |
| Platelet crit (%) | 0.23 [0.20, 0.27] | 0.24 [0.20, 0.26] | 0.25 [0.22, 0.29] | 0.25 [0.23, 0.27] |
| Platelet distribution (fL) | 11.24 [10.20, 12.50] | 11.73 [10.42, 13.30] | 11.20 [10.10, 12.17] | 11.44 [10.38, 12.90] |
| Platelet count (109/L) | 230.00 [188.00, 266.00] | 232.01 [199.00, 260.18] | 258.86 [226.50, 292.10] | 256.29 [225.00, 276.00] |
| Red blood cell count (10^12^/L) | 4.64 [4.41, 4.80] | 4.92 [4.73, 5.13] | 4.56 [4.34, 4.74] | 4.97 [4.78, 5.22] |
| RDWCV^a^ (%) | 12.30 [11.90, 12.70] | 12.40 [12.00, 12.75] | 12.50 [12.10, 12.88] | 12.50 [12.14, 12.78] |
| Total bilirubin (μmol/L) | 11.04 [10.58, 11.46] | 11.42 [11.06, 11.90] | 11.17 [9.97, 12.68] | 12.09 [10.20, 13.10] |
| Total cholesterol (mmol/L) | 4.11 [3.91, 4.26] | 4.11 [3.95, 4.24] | 4.37 [4.19, 4.54] | 4.36 [4.09, 4.57] |
| Triglycerides (mmol/L) | 0.91 [0.75, 1.11] | 1.04 [0.83, 1.21] | 1.08 [0.87, 1.21] | 1.25 [1.02, 1.35] |
| Total protein (g/L) | 70.03 [69.31, 70.70] | 70.60 [69.90, 71.79] | 73.03 [70.20, 73.32] | 73.27 [72.68, 74.20] |
| Uric acid (umol/L) | 282.53 [265.64, 318.63] | 365.00 [311.92, 400.71] | 307.86 [286.38, 339.45] | 387.13 [344.29, 423.28] |
| White blood cell count (10^9^/L) | 7.65 [6.09, 9.12] | 7.90 [6.23, 9.34] | 7.46 [5.98, 8.42] | 7.68 [6.64, 8.47] |
| urea (mmol/L) | 4.32 [3.88, 4.38] | 4.73 [4.40, 5.07] | 4.36 [4.03, 4.66] | 4.72 [4.30, 4.98] |

^a^RDWCV: red blood cell distribution width-coefficient of variation.

Moreover, we divided both the training and validation cohorts into male and female subgroups based on gender. Table S5 in Multimedia Appendix 1 shows the baseline characteristics of subjects in the subgroups. In the training cohort, subjects with PH were recorded for 3.9% (86 subjects) of females and 17.1% (299 subjects) of males, family history of hypertension for 0.7% (15 subjects) and 2.3% (41 subjects), respectively. There was also a significant difference in UA levels between males and females. These values were similar to corresponding values for both sexes in the validation cohort.

**Figure S1.** The distribution of primary hypertension in boys and girls aged 6-18 years (n=521).


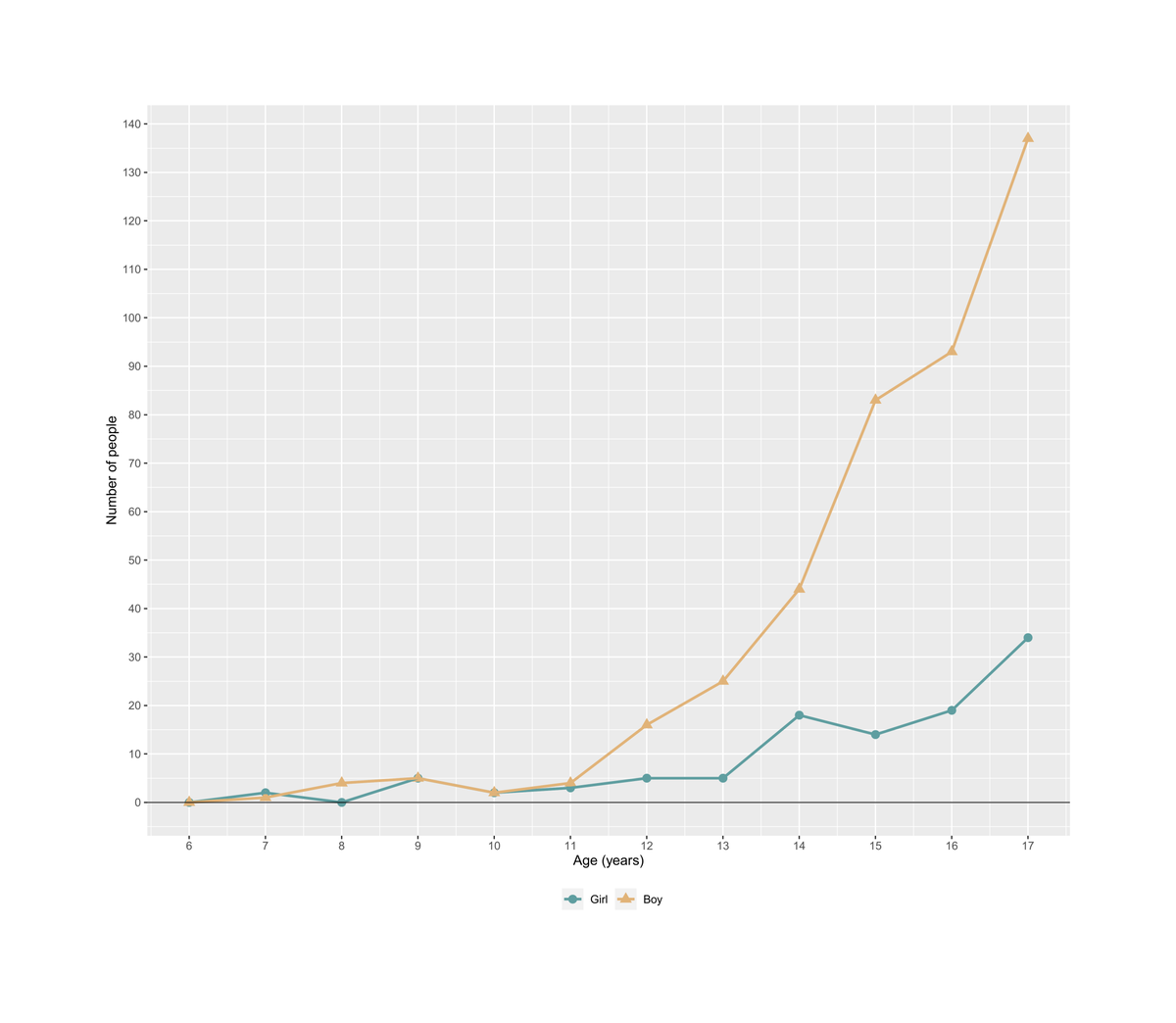


A total of 521 subjects experienced outcome events, and based on their age at onset, we found that adolescents aged 12-17 years exhibit higher prevalence rates than their younger counterparts aged 6-11 years, with a more pronounced sex-related disparity, showing a higher ratio of boys to girls (3-4:1) among adolescents with PH.
