# Supplementary material for: Development and Validation of a Nomogram-Based Model to Predict Primary Hypertension Within the Next Year in Children and Adolescents: Retrospective Cohort Study
Source: J Med Internet Res. 2024 Dec 30;26:e58686. doi: 10.2196/58686 (PMC11730233; doi:10.2196/58686)
Supplement: Multimedia Appendix 2 [file jmir_v26i1e58686_app2.docx]

**Table S1**. Baseline characteristics of individuals in the training cohort and validation cohort.

| Characteristics | | Training cohort (n=3938) | Validation cohort (n=1269) | *P* values |
| --- | --- | --- | --- | --- |
| **Diagnosis of primary hypertension, n (%)** | | | | .33 |
|  | No | 3553 (90.22) | 1133 (89.28) |  |
|  | Yes | 385 (9.8) | 136 (10.7) |  |
| **Family history of hypertension, n (%)** | | | | .58 |
|  | No | 3882 (98.58) | 1254 (98.82) |  |
|  | Yes | 56 (1) | 15 (1) |  |
| **Family history of diabetes, n (%)** | | | | .62 |
|  | No | 3923 (99.62) | 1263 (99.53) |  |
|  | Yes | 15 (0.4) | 6 (0.5) |  |
| **Breastfeeding, n (%)** | | | | <.001 |
|  | No | 2171 (55.13) | 973 (76.7) |  |
|  | Yes | 1767 (44.87) | 296 (23.3) |  |
| **Gender, n (%)** | | | | .56 |
|  | Female | 2186 (55.51) | 692 (54.5) |  |
|  | Male | 1752 (44.49) | 577 (45.5) |  |
| Age (years), median (IQR) | | 14 (11-16) | 15 (11-17) | .3 |
| White/bulb ratio (%), median (IQR) | | 1.61 (1.57-1.67) | 1.58 (1.54-1.63) | <.001 |
| Albumin (g/L), median (IQR) | | 42.72 (42.01-43.07) | 44.4 (43.76-45.26) | .08 |
| Creatine kinase (U/L), median (IQR) | | 121 (106.08-158.86) | 99.74 (85.39-109.49) | <.001 |
| Calcium (mmol/L), median (IQR) | | 2.35 (2.23-2.44) | 2.37 (2.31-2.44) | .12 |
| Creatinine (umol/L), median (IQR) | | 46.38 (38.51-62.29) | 59.5 (47.93-69.52) | <.001 |
| Direct bilirubin (μmol/L), median (IQR) | | 3.88 (3.53-4.2) | 4.22 (3.55-4.89) | <.001 |
| Fasting blood glucose (mmol/L), median (IQR) | | 4.92 (4.85-4.99) | 5.09 (4.86-5.16) | .1 |
| Globulin (g/L), median (IQR) | | 27.63 (26.53-27.99) | 28.67 (27.2-29.04) | <.001 |
| Hematocrit (%), mean (SD) | | 0.42 (0.03) | 0.42 (0.03) | .62 |
| High-density lipoprotein cholesterol (mmol/L), median (IQR) | | 1.23 (1.14-1.32) | 1.25 (1.11-1.39) | <.001 |
| Hemoglobin (g/L), median (IQR) | | 135.37 (129-144.78) | 136.52 (129.45-145) | .56 |
| Indirect bilirubin (μmol/L), mean (SD) | | 7.19 (1.05) | 7.2 (1.64) | .7 |
| Kalium (mmol/L), median (IQR) | | 4.18 (4.11-4.26) | 4.14 (4.06-4.21) | <.001 |
| Lactate dehydrogenase (U/L), median (IQR) | | 224.72 (202.6-245.99) | 200.91 (175.59-224) | <.001 |
| Low-density lipoprotein cholesterol (mmol/L), median (IQR) | | 2.65 (2.46-2.72) | 2.67 (2.55-2.8) | .43 |
| Lipoprotein(a) (mg/L), median (IQR) | | 183.7 (152.89-193.2) | 180.53 (101-180.66) | <.001 |
| Lymphocyte count (10^9^/L), mean (SD) | | 2.16 (0.85) | 2.15 (0.74) | .84 |
| Lymphocyte percentage (%), mean (SD) | | 29.41 (11.1) | 30.02 (9.67) | .08 |
| Mean corpuscular hemoglobin (pg), median (IQR) | | 28.65 (27.8-29.4) | 28.96 (28.31-29.7) | <.001 |
| Mean cell hemoglobin concentration (g/L), median (IQR) | | 328.61 (316-339) | 330.49 (326.33-335) | <.001 |
| Mean corpuscular volume (fL), mean (SD) | | 87.58 (4.12) | 87.58 (3.24) | .99 |
| Monocyte count (10^9^/L), median (IQR) | | 0.6 (0.47-0.68) | 0.57 (0.46-0.64) | .76 |
| Monocyte percentage (%), median (IQR) | | 7.8 (6.47-10) | 7.54 (6.4-8.8) | <.001 |
| Mean platelet volume (fL), median (IQR) | | 10.2 (9.7-10.7) | 9.89 (9.4-10.4) | <.001 |
| Neutrophil count (10^9^/L), median (IQR) | | 4.71 (3.27-6.09) | 4.7 (3.48-5.3) | 0.005 |
| Neutrophil percentage (%), mean (SD) | | 59.95 (11.89) | 59.42 (10.28) | .15 |
| Natrium (mmol/L), median (IQR) | | 138.35 (137.62-139.26) | 138.9 (138.03-139.85) | <.001 |
| Plateletcrit (%), median (IQR) | | 0.23 (0.2-0.26) | 0.25 (0.23-0.28) | <.001 |
| Platelet distribution (fL), mean (SD) | | 11.65 (1.81) | 11.55 (1.8) | .1 |
| Platelet count (109/L), median (IQR) | | 231 (194-264) | 257.63 (225-285) | <.001 |
| Red blood cell count (10^12^/L), mean (SD) | | 4.75 (0.37) | 4.76 (0.41) | .84 |
| Red blood cell distribution width-coefficient of variation (%), median (IQR) | | 12.34 (11.93-12.73) | 12.5 (12.1-12.8) | <.001 |
| Total bilirubin (μmol/L), median (IQR) | | 11.24 (10.73-11.61) | 11.58 (10.1-12.86) | <.001 |
| Total cholesterol (mmol/L), median (IQR) | | 4.11 (3.92-4.25) | 4.37 (4.14-4.55) | .05 |
| Triglycerides (mmol/L), median (IQR) | | 1.10 (0.88-1.27) | 1.15 (0.9-1.28) | .31 |
| Total protein (g/L), median (IQR) | | 70.27 (69.31-71.04) | 73.12 (72.01-73.56) | <.001 |
| Uric acid (umol/L), median (IQR) | | 340 (293.83-369.55) | 338.29 (296.91-389.62) | .08 |
| White blood cell count (10^9^/L), median (IQR) | | 7.74 (6.14-9.22) | 7.64 (6.31-8.46) | 0.001 |
| Urea (mmol/L), median (IQR) | | 4.37 (4.06-4.8) | 4.5 (4.13-4.87) | .06 |
